# mRNA and long non-coding RNA expression profiles of rotator cuff tear patients reveal inflammatory features in long head of biceps tendon

**DOI:** 10.1186/s12920-022-01292-y

**Published:** 2022-06-20

**Authors:** Yi-Ming Ren, Yuan-Hui Duan, Yun-Bo Sun, Tao Yang, Wei-Yu Hou, Chang Liu, Meng-Qiang Tian

**Affiliations:** 1 Department of Joint and Sport Medicine, Tianjin Union Medical Center, Nankai University Affiliated People’s Hospital, Jieyuan Road 190, Hongqiao District, Tianjin, 300121 People’s Republic of China; 2 Schoole of Medicine, Nankai University, Tianjin, People’s Republic of China

**Keywords:** Long head of biceps tendon, Rotator cuff, Inflammation, Long non-coding RNA

## Abstract

**Background:**

This study aimed to identify the differentially expressed mRNAs and lncRNAs in inflammatory long head of biceps tendon (LHBT) of rotator cuff tear (RCT) patients and further explore the function and potential targets of differentially expressed lncRNAs in biceps tendon pathology.

**Methods:**

Human gene expression microarray was made between 3 inflammatory LHBT samples and 3 normal LHBT samples from RCT patients. GO analysis and KEGG pathway analysis were performed to annotate the function of differentially expressed mRNAs. The real-time quantitative reverse transcription-polymerase chain reaction (qRT-PCR) was admitted to verify their expression. LncRNA-mRNA co-expression network, cis-acting element, trans-acting element and transcription factor (TF) regulation analysis were constructed to predict the potential molecular regulatory ﻿mechanisms and targets for LHB tendinitis.

**Results:**

103 differentially expressed lncRNAs and mRNAs, of which 75 were up-regulated and 28 were down-regulated, were detected to be differentially expressed in LHBT. The expressions of 4 most differentially expressed lncRNAs (A2MP1, LOC100996671, COL6A4P, lnc-LRCH1-5) were confirmed by qRT-PCR. GO functional analysis indicated that related lncRNAs and mRNAs were involved in the biological processes of regulation of innate immune response, neutrophil chemotaxis, interleukin-1 cell response and others. KEGG pathway analysis indicated that related lncRNAs and mRNAs were involved in MAPK signaling pathway, NF-kappa B signaling pathway, cAMP signaling pathway and others. TF regulation analysis revealed that COL6A4P2, A2MP1 and LOC100996671 target NFKB2.

**Conclusions:**

LlncRNA-COL6A4P2, A2MP1 and LOC100996671 may regulate the inflammation of LHBT in RCT patients through NFKB2/NF-kappa B signaling pathway, and preliminarily revealed the pathological molecular mechanism of tendinitis of LHBT.

**Supplementary Information:**

The online version contains supplementary material available at 10.1186/s12920-022-01292-y.

## Introduction

Chronic rotator cuff tears (RCTs) commonly contribute to shoulder pain and may be caused by tendinopathy of the long head of the biceps tendon (LHBT). In particular, LHBT pathology may be a generally overlooked cause of persistent anterior shoulder pain after rotator cuff repair. Clinically, the damage of LHBT is often used in combination with RCT, but it rarely occurs alone [1–4]. Many studies have proven that a higher incidence of macroscopic pathological changes accompany LHBT in RCT, especially in large tears [3, 5–7]. In addition, several situations have been described in which LHBT spontaneously ruptures after insertion, which can significantly relieve pain and improve function on the shoulders of RCTs receiving conservative treatment [8]. Challenges still exist about how to manage concomitant long head of biceps tendinopathy during RCT repair surgery. Even controversy persists in the literature regarding the function of the LHBT and the pathological mechanism of its disorders. Some researchers believe that LHBT tendinitis occurs as inflammatory tenosynovitis of the tendon course along the restraint path in the biceps groove of the humerus [9, 10]. Murthi hold that this long head of bicep tendinopathy is characterized by chronic inflammatory process, overuse and decreased tenoblastic capacity associated with increasing release of neurotransmitters such as calcitonin gene related peptide and P substance [4]. Nevertheless, like achilles tendinitis and others, the pathophysiological manifestations of tendinitis are mechanical overload, tissue inhibitor of metalloproteinase, imbalance between matrix metalloproteinase and inflammation, and imbalance of cell apoptosis [11–14].

Long non-coding RNA (lncRNA) that is over 200 nucleotides for length is defined as a transcript that is not translated into protein [15]. LncRNA has been determined to have functional roles in a variety of cell functions, such as differentiation, development, cell fate, and disease pathogenesis [16]. In recent years, lncRNAs have been widely studied, and more and more evidences show that they appear as key and indispensable transcriptional and post-transcriptional mediators in various physiological and pathological processes in a tissue-specific manner [17–19]. To date, some histopathology researches of the LHBT were made to try to clarify the pathological mechanisms of the LHB tendinitis [20–22]. Some researchers believe that CD44 may affect LHB tendinopathy by inflammation, regulating apoptosis, and extracellular matrix homeostasis [23]. Whether the lncRNAs also play a role in the tendinopathy of LHBT caused by chronic inflammation remains to be explored.

﻿As such, this study was undertaken to elucidate the differentiation expressed mRNAs and lncRNAs in inflammatory LHBT of RCT patients and further probe the function and potential targets of differentiation expressed lncRNAs in biceps tendon pathology. ﻿We hypothesized that molecular biological changes will appear between the inflammatory LHBT samples and normal LHBT samples, and the lncRNAs and relative mRNAs might play roles in inflammatory features of LHBT.

## Materials and methods

### Patients and tissue samples collection

**Fig. 1:**
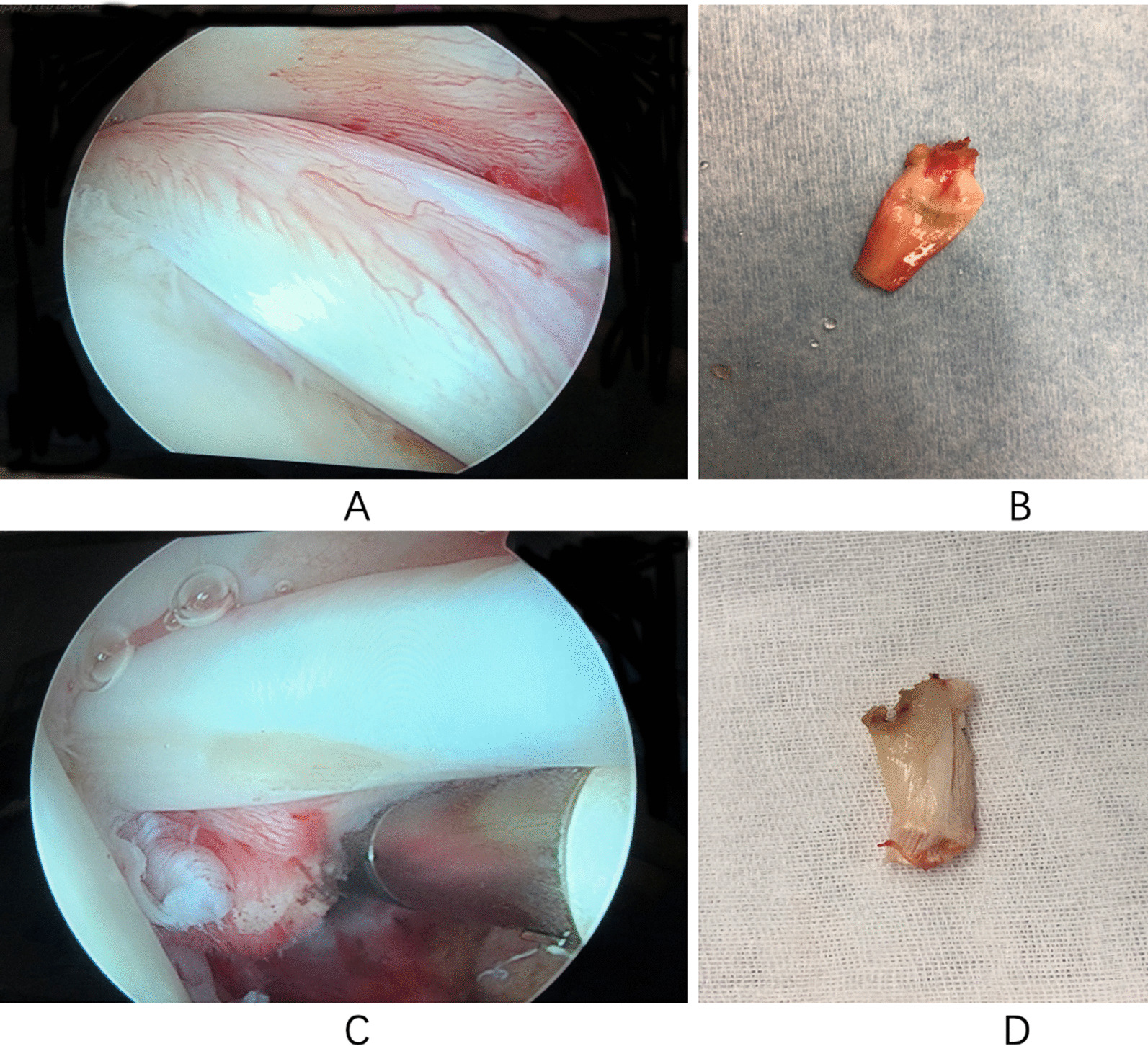
Macroscopic and microscopic view of inflamed and non-inflamed tendon samples. long head of the biceps tendon (LHBT) samples with (**A**, **B**) and without (**C**, **D**) tendinitis are shown. **A**, **C** Intraoperative arthroscopic view. **B**, **D** Tendon samples before processing in the laboratory.

The patients with traumatic RCTs were middle-aged people. The rotator cuff and LHBT were suddenly broken. The tendon tissues were healthy without obvious degeneration and inflammation. However, all patients with degenerative RCTs were elderly. The rupture of rotator cuff and LHBT was caused by chronic wear and impact. Tendons often have obvious acute or chronic inflammation. LHBT with clear macroscopic signs of inflammation was collected from 8 degenerative RCT shoulders (mean donor age 63.1 years (52–69 years), including 3 males and 5 females’ patients), and 8 traumatic normal LHBTs were collected from the RCT shoulder (mean donor age 52.0 years (44–59 years), including 4 males and 4 females’ patients). All patients underwent an arthroscopic-assisted biceps tenodesis. The intra-articular portion of LHBT tendons was obtained from each of the patients who underwent arthroscopy. Among them, 3 inflammatory LHBT samples and 3 traumatic normal LHBT samples were used for gene expression microarray. 3 inflammatory LHBT samples and 3 traumatic normal LHBT samples were used for quantitative real-time PCR assay. 2 inflammatory LHBT samples and 2 traumatic normal LHBT samples were discarded because they did not meet the conditions of the chip experiment. As shown in Fig. 1, the macro images of all samples were taken from arthroscopy. The tendon samples were collected and fixed in 4% paraformaldehyde (Gibco, USA) and 0.1 M phosphate buffer solution (PBS, Gibco, USA) at pH 7.4 until 24 hours at 4 °C. Thereafter, the tissues were rinsed in PBS at pH 7.4, frozen embedded and kept at − 80 °C. All procedures of this research were approved by the local Ethics Committee for Research on Human Beings of Tianjin Union Medical Center (2021-SYDWLL-000178). All patients voluntarily agreed to participate and freely signed an informed consent form. All methods were performed in accordance with the relevant guidelines and regulations.

### RNA isolation, library preparation and sequencing

In this study, Agilent SurePrint G3 human gene expression microarray (v3 8x60K, DesignID: 072363) was used. The data analysis of these 6 samples was handled by OE Biotechnology Co., Ltd. (Shanghai, China). In brief, NanoDrop ND-2000 (Thermo Scientific, USA) quantified all RNA of each sample, and Agilent Bioanalyzer-2100 (Agilent Technologies, USA) assessed the RNA integrity. All procedures including sample labeling, washing and microarray hybridization, were handled basing the manufacturer's proposals. The all RNA was transcribed into double-stranded cDNA, and furtherore synthesized into cRNA with Cyanine-3-CTP labeled. After washing with PBS and hybridized onto the microarray, Agilent Scanner-G2505C (Agilent Technologies, USA) canned the arrays.

### Data acquisition and bioinformatics analysis

For bioinformatics analysis, all original data were imported and analyzed with Feature Extraction v10.7.1.1 (Agilent Technologies, USA) and GeneSpring v14.8 (Agilent Technologies, USA). After normalizing all the data, the differentiation expressed genes are screened out according to *P* value (T-Test, fold change ≥ 2.0, along with *P* ≤ 0.05). In addition, Gene Ontology (GO) analysis and Kyoto Encyclopedia of Genes and Genome (KEGG) analysis [24] could determine the distinguishable genes' expression pattern and roles among these differentially expressed mRNAs.

### Construction of lncRNA-mRNA co-expression network

Basing on the standardized signal intensity of specifically lncRNAs and mRNAs, the co-expression network was established. LncRNA-mRNA with Pearson correlation coefficient value ≥ 0.8 along with *P* ≤ 0.05 were included. Moreover, differentiation expressed mRNAs among the target genes of mRNAs were imported and analyzed with Cytoscape software (v3.6.1).

### Target gene analysis of lncRNA Cis-acting element and trans-acting element

The effect of lncRNA on neighboring target genes was usually called cis role. When knowing results of gene co-expression, FEELnc software was used to search for total of the coding genes within 100kb upstream and downstream of differentially expressed lncRNAs. Then the differentiation expressed genes with significant co-expression (Pearson’s correlation calculation) with the lncRNAs were intersected. These genes are near the genome and co-expressed in the expression pattern, which is likely to be regulated by these lncRNAs.

The trans role means the lncRNAs could affect other genes at the expression level. According to gene co-expression results, RNA interaction software RIsearch-2.0 could predict the binding of candidate co-expressed lncRNAs and genes at the nucleic acid level. Direct regulation exist between the screened lncRNAs and genes, when screening condition satisfied the base number of direct interaction between two nucleic acid molecules is ≥ 10, and the base binding free energy is ≤ − 100.

### Correlation analysis of lncRNA transcription factors

Basing gene co-expression results, each differentiation expressed lncRNA and their co-expressed coding genes, as well as the differential gene enrichment significance in each transcription factor (TF) entry are confirmed by calculation by clusterprofiler R software package via the gene TF relationship pairs provided by Gene Transcription Regulation Database (http://gtrd.biouml.org).

### Quantitative real-time PCR assay

All RNA was collected by extracting six different tissues using TRIzol (Ambion, USA). 1 microgram of all RNA every sample were reverse transcribed using the HiScript® II Q RT SuperMix qPCR (+gDNA wiper) (VAZYME, China). In addition, the thermal program underwent 10 min at 95 °C, then 40 cycles of amplifications, 15 s at 95 °C for denaturation, 60 s at 60 °C for annealing, and 15 s at 95 °C for extension. Total of samples were carried out on QuantStudio 6 Thermal Cycler (ABI, USA) using SYBR Green PCR Master Mix (VAZYME, China). An internal control including GAPDH and β-actin, and the primers are shown in Additional file 1. Each sample was analyzed and calculated in triplicate. The 2^−ΔΔCt^ method was used to calculate the relative quantification of the interested hub genes [25].

## Results

### Expression profiles of mRNAs and lncRNAs in LBHT

**Fig. 2:**
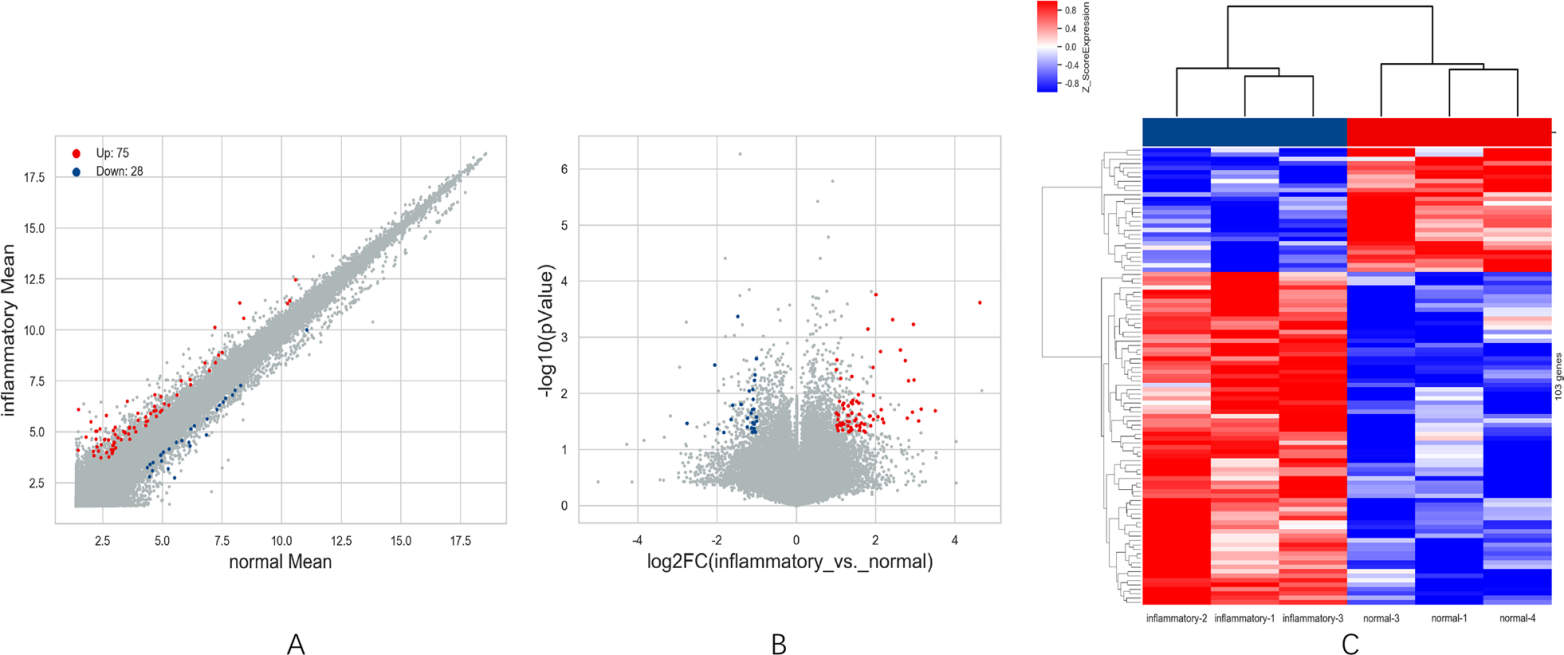
Scatter plot (**A**), Volcano plots (**B**) and heat map (**C**) showing expression profiles of long non-coding RNAs (lncRNAs) and mRNAs in inflammatory and normal long head of biceps tendon (LHBT). Three plots are based on the expression values of all lncRNAs and mRNAs detected by microarray. These maps showing significantly changed lncRNAs and mRNAs with fold change ≥ 2.0 respectively (*P* < 0.05; false discovery rate < 0.05).

**Fig. 3:**
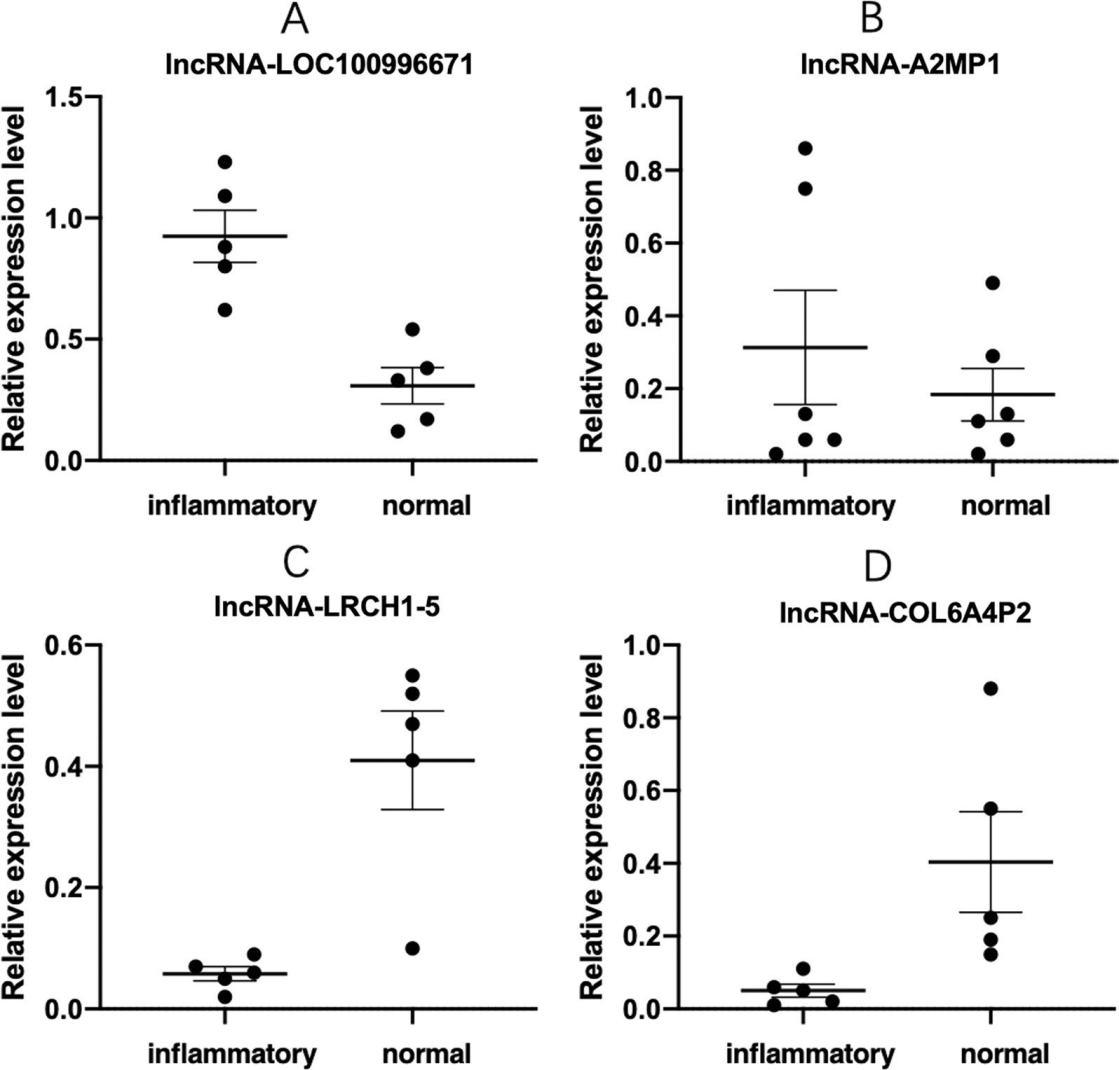
qRT-PCR validation. qRT-PCR verification of 4 candidate lncRNAs in 3 pairs of inflammatory and normal LHBT tissue. Expression of inflammatory vs. normal samples was analyzed using qRT-PCR, and summarized as mean average ± standard error (SE). *P* < 0.05 was considered statistically significant.

Over 6400 lncRNAs and 43 differentiation expressed lncRNAs were discovered basing on the whole expression profile, including 31 upregulated lncRNAs (such as LOC100996671, A2MP1, LINC01574, LOC102725470 and others) and 12 down-regulated ones (lnc-LRCH1-5, lnc-MTERF-5, COL6A4P2, lnc-RPL31-1 and others) in LHBT. 9 down-regulated mRNAs (SLC5A11, TGM6, OVOS2, OR10A5 and others) and 37 up-regulated mRNAs (such as SLC7A14, HCAR3, C4BPA, S100A12 and others) were detected from 46 differentially expressed mRNAs in LHBT (*p *< 0.05, along with fold change ≥ 2, Fig. 2A–C). Among them, the top 10 differentiation expressed lncRNAs and mRNAs were shown in Tables 1 and 2. Among those differentiation expressed lncRNAs, several thoroughly studied molecules including A2MP1, C1QTNF1-AS1, CASC2, FTCDNL1, FTX, LOC339975 and TWIST1, which expressed in other diseases, were also significantly changed in LHBT. Thus, 4 significantly expressed lncRNAs (A2MP1, LOC100996671, COL6A4P, lnc-LRCH1-5) were further validated by qRT-PCR (Fig. 3A–D). LOC100996671 (Fig. 3A) and A2MP1 (Fig. 3B) were upregulated, and lnc-LRCH1-5 (Fig. 3C) and COL6A4P2 (Fig. 3D) were ﻿downregulated in LHBT. ﻿The results of qRT-PCR showed no difference with the expression changes of these four genes in previous microarray. All 4 lncRNAs were differentially expressed (up-regulated or down-regulated) with the same trend.

**Table 1:** Top 20 aberrantly expressed lncRNAs in microarray for 3 pairs of inflamed and non-inflamed LHBT samples

ProbeName	pValue	log2FC	Regulation	GeneSymbol	Accession
A_22_P00005144	0.02048574	3.49963572	Up	LOC100996671	NR_110035
A_21_P0011082	0.00058948	2.95327361	Up	A2MP1	NR_040112
A_21_P0004343	0.0059595	2.83196966	Up	LINC01574	NR_108030
A_21_P0005162	0.02773331	2.79987563	Up	LOC102725470	XR_426000
A_21_P0001849	0.00048847	2.4311316	Up	LINC00486	NR_027099
A_22_P00007566	0.00178438	2.12452699	Up	lnc-HBZ-1	lnc-HBZ-1:1
A_22_P00006369	0.02617198	2.05825362	Up	LOC339975	NR_038931
A_21_P0010423	0.00017424	2.00894029	Up	lnc-TBC1D22A-1	lnc-TBC1D22A-1:2
A_21_P0008063	0.04965323	− 1.8317208	Down	lnc-LRCH1-5	–
A_22_P00010253	0.0294142	− 1.6445104	Down	lnc-MTERF-5	–
A_21_P0012395	0.0161794	− 1.6103221	Down	COL6A4P2	NR_027898
A_22_P00013849	0.04200824	− 1.1273853	Down	lnc-RPL31-1	–
A_22_P00013643	0.0492766	− 1.1078939	Down	LOC101928785	XR_245261
A_22_P00005665	0.0473815	− 1.0669176	Down	TUB-AS1	NR_120537
A_22_P00014771	0.04257993	− 1.0625811	Down	lnc-SLC36A4-1	–
A_24_P233078	0.00592137	− 1.0547137	Down	PYY2	NR_003064
A_21_P0012241	0.00465434	− 1.0373481	Down	LOC102725394	XR_424435
A_22_P00018036	0.0316348	− 1.0089437	Down	lnc-ZNF236-1	–
A_21_P0000379	0.02650708	− 1.0072159	Down	SNORD67	NR_003056
A_21_P0008063	0.04965323	− 1.8317208	Down	lnc-LRCH1-5	XR_249342

LHBT, long head of the biceps tendon; FC, fold change.

**Table 2:** Top 20 aberrantly expressed mRNAs in microarray for 3 pairs of inflamed and non-inflamed LHBT samples

ProbeName	pValue	log2FC	Regulation	GeneSymbol	Accession
A_22_P00002897	0.02942744	1.94025954	Up	APELA	–
A_23_P97541	0.03095731	3.08205494	Up	C4BPA	NM_000715
A_23_P64721	0.01906914	3.14941279	UP	HCAR3	NM_006018
A_33_P3273884	0.02866454	2.17088461	Up	HLA-DQA1	NM_002122
A_23_P137484	0.01950713	2.13576295	Up	L1TD1	NM_019079
A_23_P251151	0.00168409	2.62172293	Up	NELL1	NM_006157
A_33_P3385785	0.00259619	2.74783197	Up	S100A12	NM_005621
A_23_P74001	0.00578642	2.96591318	Up	S100A12	NM_005621
A_23_P253451	0.00024083	4.62521439	Up	SLC7A14	NM_020949
A_33_P3273885	0.02181321	2.93037197	Up	–	XM_005274953
A_22_P00002897	0.02942744	1.94025954	Up	APELA	–
A_23_P170534	0.03537931	− 1.0683069	Down	FUT7	NM_004479
A_24_P150791	0.01915975	− 1.0710058	Down	JPH3	NM_020655
A_22_P00009331	0.04934704	-1.0327538	Down	LRRC7	–
A_32_P105825	0.02744632	− 1.2323383	Down	MPPED2	NM_001584
A_23_P315991	0.03978925	− 1.2392088	Down	OR10A5	NM_178168
A_33_P3416797	0.00042737	− 1.4783867	Down	OVOS2	–
A_23_P115467	0.02275422	− 1.1365521	Down	S100A5	NM_002962
A_23_P37914	0.00312407	− 2.0578847	Down	SLC5A11	NM_052944
A_33_P3249259	0.0431449	− 1.9907882	Down	TGM6	NM_198994

LHBT, long head of the biceps tendon; FC, fold change.

### GO annotation and KEGG enrichment analysis

**Fig. 4:**
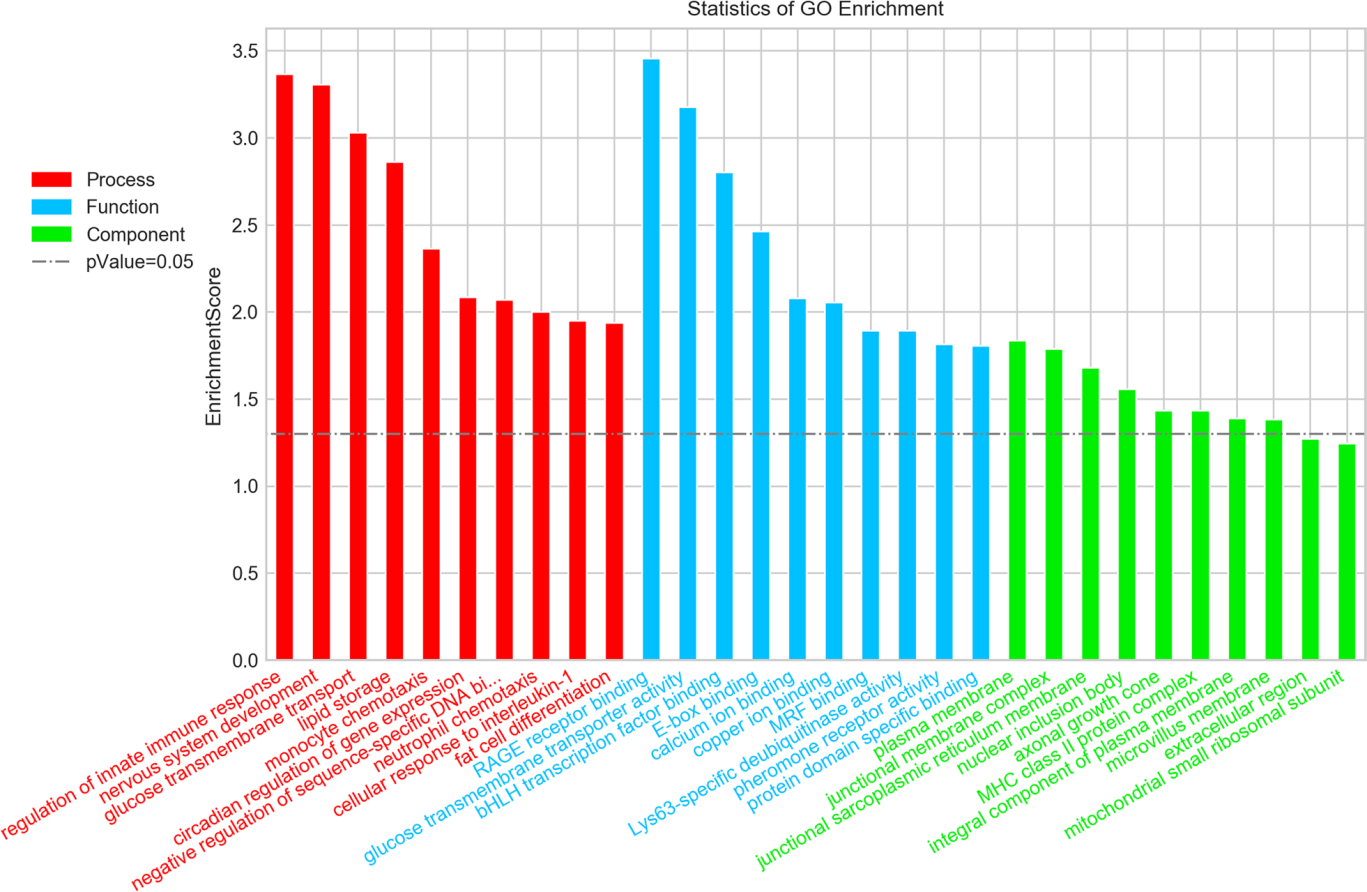
Gene Ontology (GO) annotations of up-and down regulated lncRNAs and mRNAs (fold change > 2; *p* < 0.05) with top 10 enrichment scores of biological processes. This bar chart shows the go items of top 10 in x-axis. Y axis called enrichment score is − log10 (*p* value), and the higher the bar graph height, the smaller the corresponding *p* value. Different color distributions correspond to cellular component (green), biological process (red) and molecular function (blue).

There were 74 genes were annotated by ﻿using the GO database, including 40 genes annotated by biological process (BP), 42 genes annotated by cell component (CC), and 42 genes annotated by molecular function (MF) (Fig. 4). Fisher's accurate test is used to calculate the enrichment significance of each term in BP, CC and MF respectively. Each term is arranged in ascending order on account of P value, that is, the more significant the enrichment (the smaller the P value), the higher the enrichment. There are 114 terms along with *P* ≤ 0.05. The first three terms with the smallest *P* value are RAGE receptor binding (TermID:GO:0050786, *P* value: 0.00035); regulation of innate immune response (TermID:GO:0045088, *P* value: 0.00043); nervous system development (TermID:GO:0007399, *P* value: 0.0005); and 3 terms with false discovery rate (FDR) ≤ 0.05. There are 80 terms along with *P* ≤ 0.05 in BP, 0 term with FDR ≤ 0.05, 8 terms along with *P* ≤ 0.05 in CC, 0 term with FDR ≤ 0.05, 26 terms with *P* ≤ 0.05 as well as 3 terms with FDR ≤ 0.05 in MF.

**Fig. 5:**
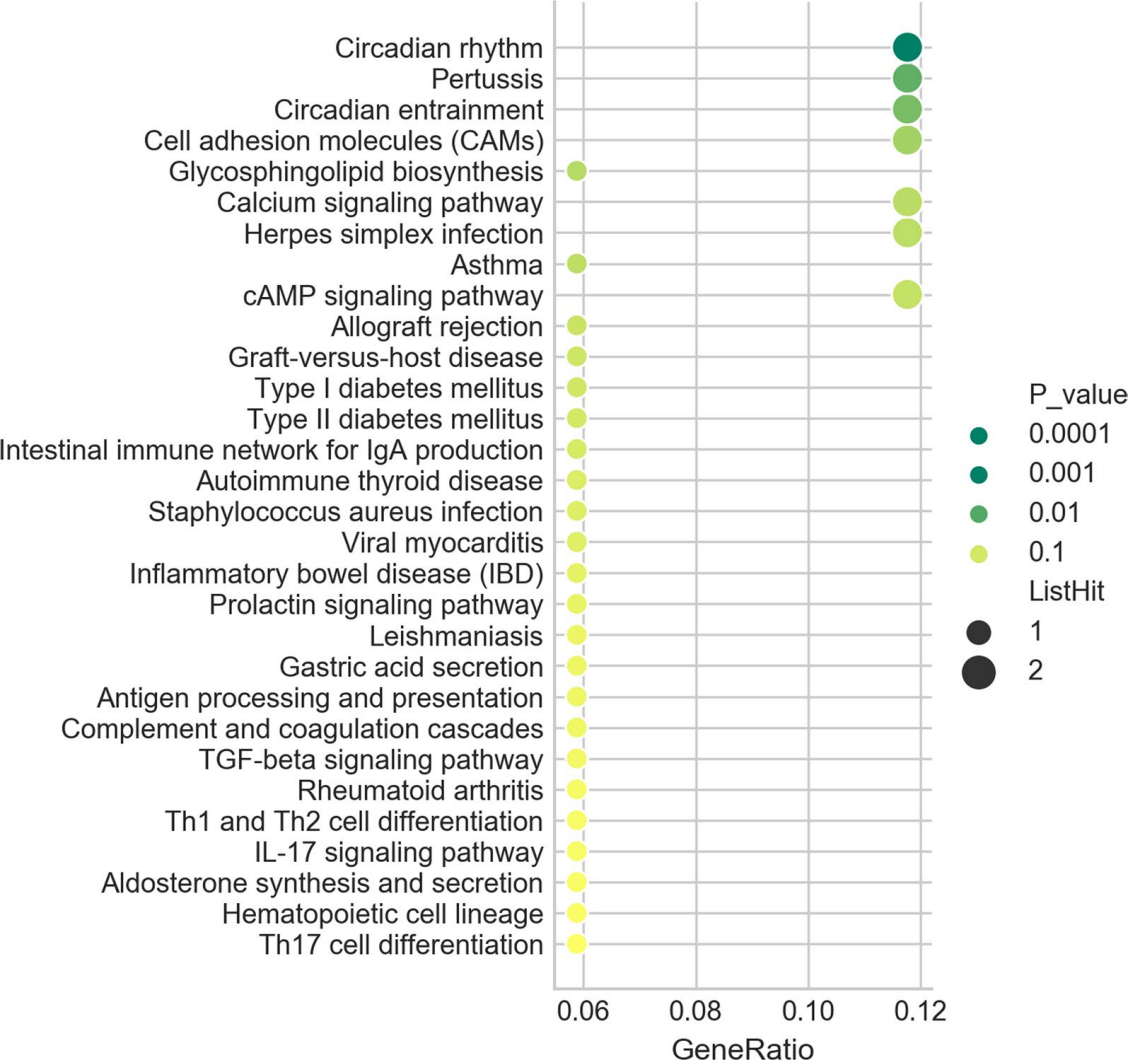
Kyoto Encyclopedia of Genes and Genomes ﻿(KEGG) pathway enrichment analysis scores of up-and down regulated lncRNAs and mRNAs with top 30. This bubble chart shows that the x-axis called gene ratio represents the enrichment degree and the y-axis represents the enrichment pathway. The larger the circle dot is, the more genes fall into the pathway. The greener the color is, and the higher the significance of enrichment is.

There were 74 genes in total, 17 of which were annotated to KEGG pathway. The hypergeometric distribution was used to calculate the correlation between each pathway in KEGG pathway and the differentially expressed gene. Each pathway is arranged in ascending order on account of P value, and the more significant the enrichment is (the smaller the *P* value is), the more advanced it is. Among them, there are 4 pathways with *P* ≤ 0.05, and the first 3 pathways with *P* value minimum respectively are Circadian rhythm (TermID:path:hsa04710, *P* value:0.0023); Pertussis (TermID:path:hsa05133, *P* value:0.013); Circadian entrainment (TermID:path:hsa04713, *P* value:0.02); and 0 pathway with FDR ≤ 0.05. All 17 pathways were presented in Fig. 5 [24].

### Co‑expression network of ﻿lncRNA-mRNA

**Fig. 6:**
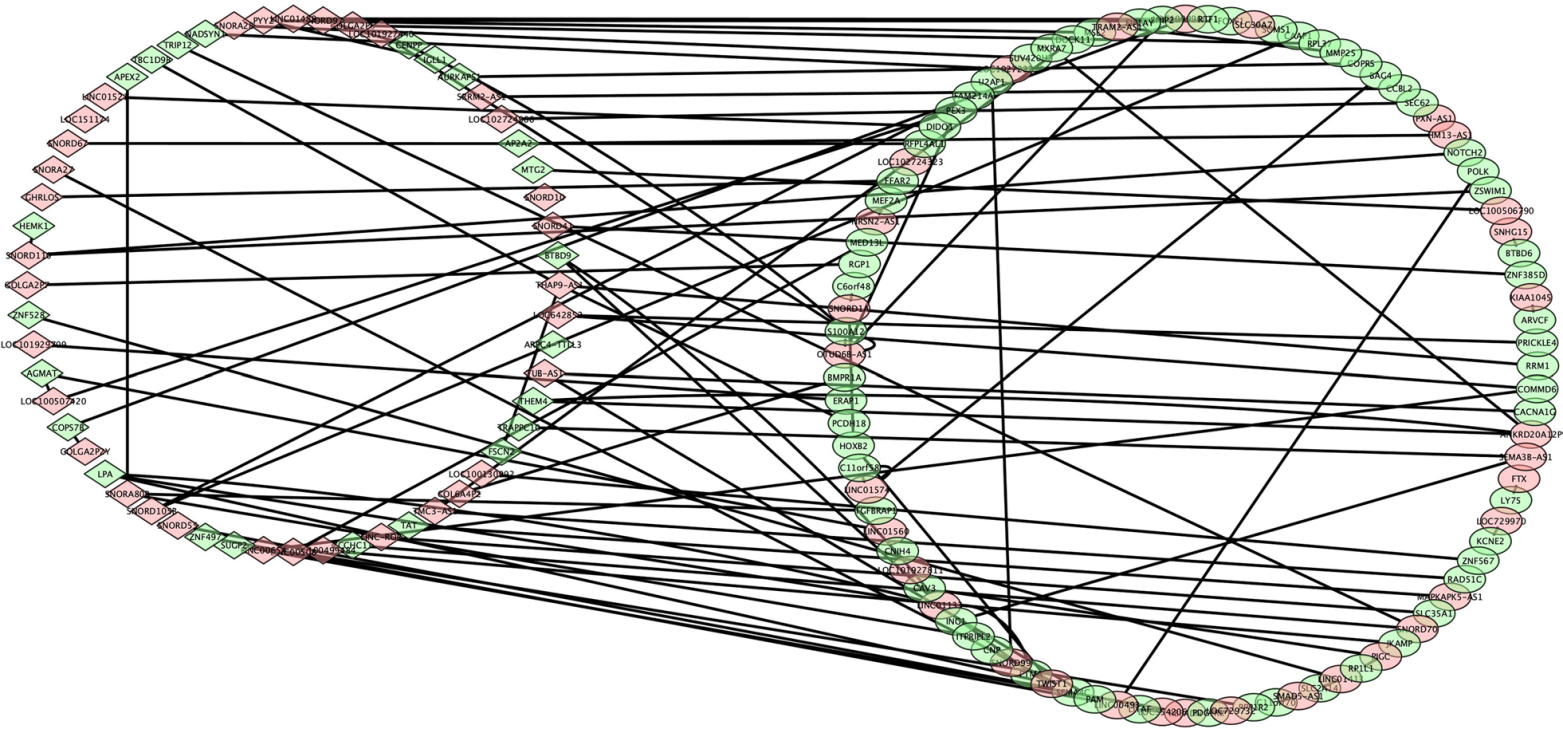
The lncRNA-mRNA co-expression network with significant values of Pearson correlation coefficients (*p *< 0.05). The rhombuses denote lncRNAs and the ellipses denote mRNAs (green: downregulated genes; red: upregulated genes). An edge represents a co-expression relationship between mRNA and a lncRNA in the development of LHB tendinitis. Data were analyzed and constructed by Cytoscape software. This co-expression network suggests an inter-regulation of lncRNAs and mRNAs in LHB tendinitis.

According to the data of mRNA-lncRNA co-expression network which we structured, 32631 lncRNA-mRNA pairs (including repeated pairs) with significant Pearson correlation coefficient values (*p* < 0.05) were selected. In addition, 65 remarkable expressed lncRNAs and 87 remarkable expressed mRNAs of our data were selected to contribute to a network diagram containing 102 associations (Fig. 6). This network showed the overall prospect of the complex regulatory relationship among lncRNA and mRNA during the pathological change of LHB tendinitis. In this network, different lncRNAs can regulate one mRNA, and one lncRNA can regulate different mRNAs. The multilateral interaction between lncRNA and mRNA forms a complex regulatory mechanism.

### Cis and trans role of lncRNAs

**Fig. 7:**
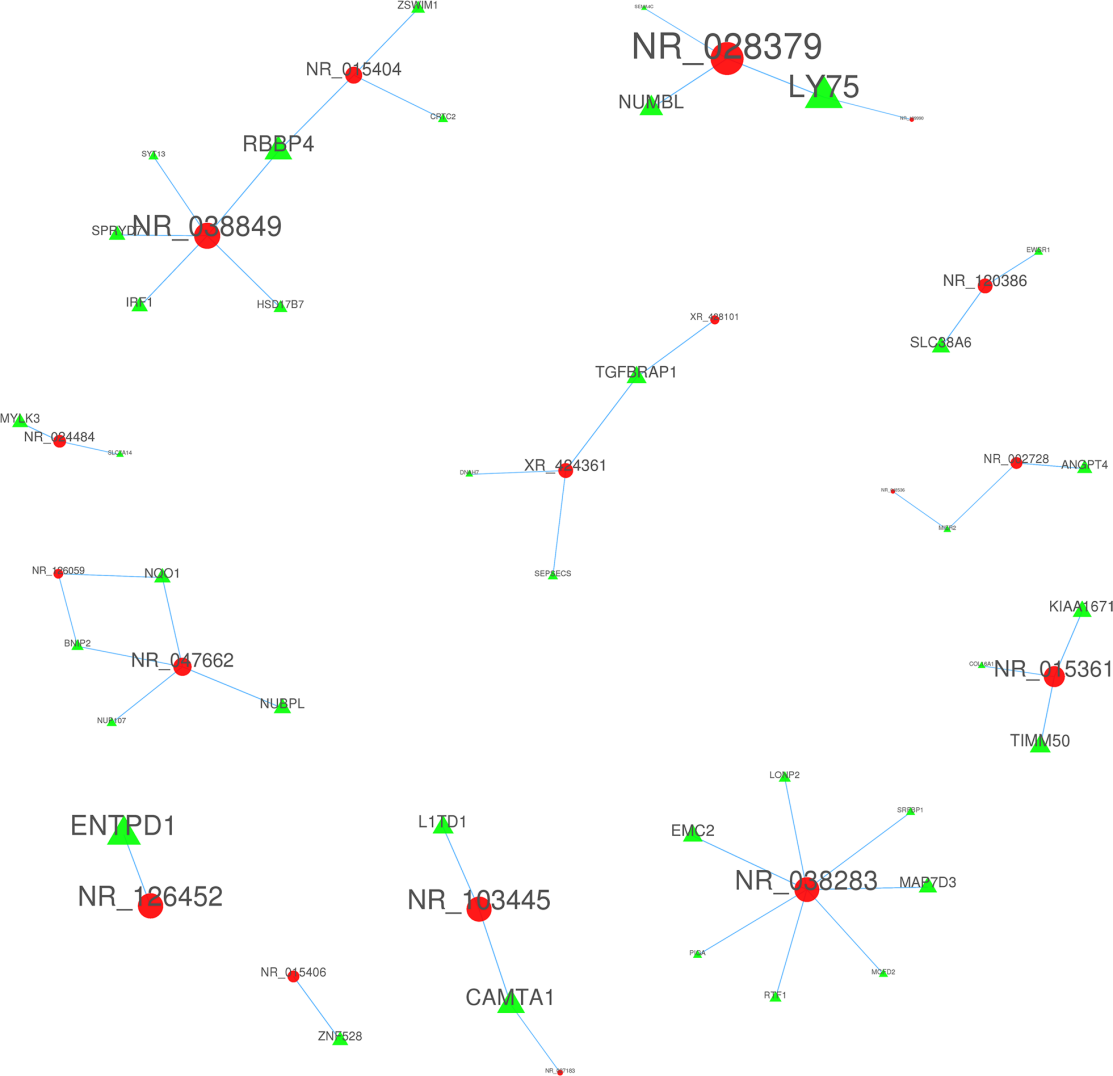
Networks analysis of putative interactions between 18 selected lncRNAs and trans-regulated mRNAs related to the tendinopathy of LHBT. Circular (red) and triangular (green) nodes represent lncRNAs and protein-coding genes, respectively.

By predicting the potential cis and trans targets of lncRNAs, we tried to dig the functions of Top 500 differentially expressed lncRNAs. ﻿Regarding cis action, Top 500 differentially expressed lncRNAs corresponded to 0 protein-coding gene. ﻿As fig. 7 shown, a total of 18 transactions between lncRNAs and protein-coding genes were identified, as well as all 44 interactions were identified. The interactive networks are quite complex, and there are obviously some anti-regulatory relationships. For example, some mRNAs (RBBP4, LY75, TGFBRAP1, MIER2, NQO1, BNIP2, CAMTA1) can be regulated by different lncRNAs (LINC01133(NR_038849), MAPKAPK5-AS1(NR_015404), FTX(NR_028379), NRSN2-AS1(NR_109990) and others), and one lncRNA (PTPRG-AS1(NR_038283)) can target many mRNAs (MCFD2, RTF1, LONP2, SRFBP1, MAP7D3, EMC2 and PIGA).

### TFs role of lncRNAs

**Fig. 8:**
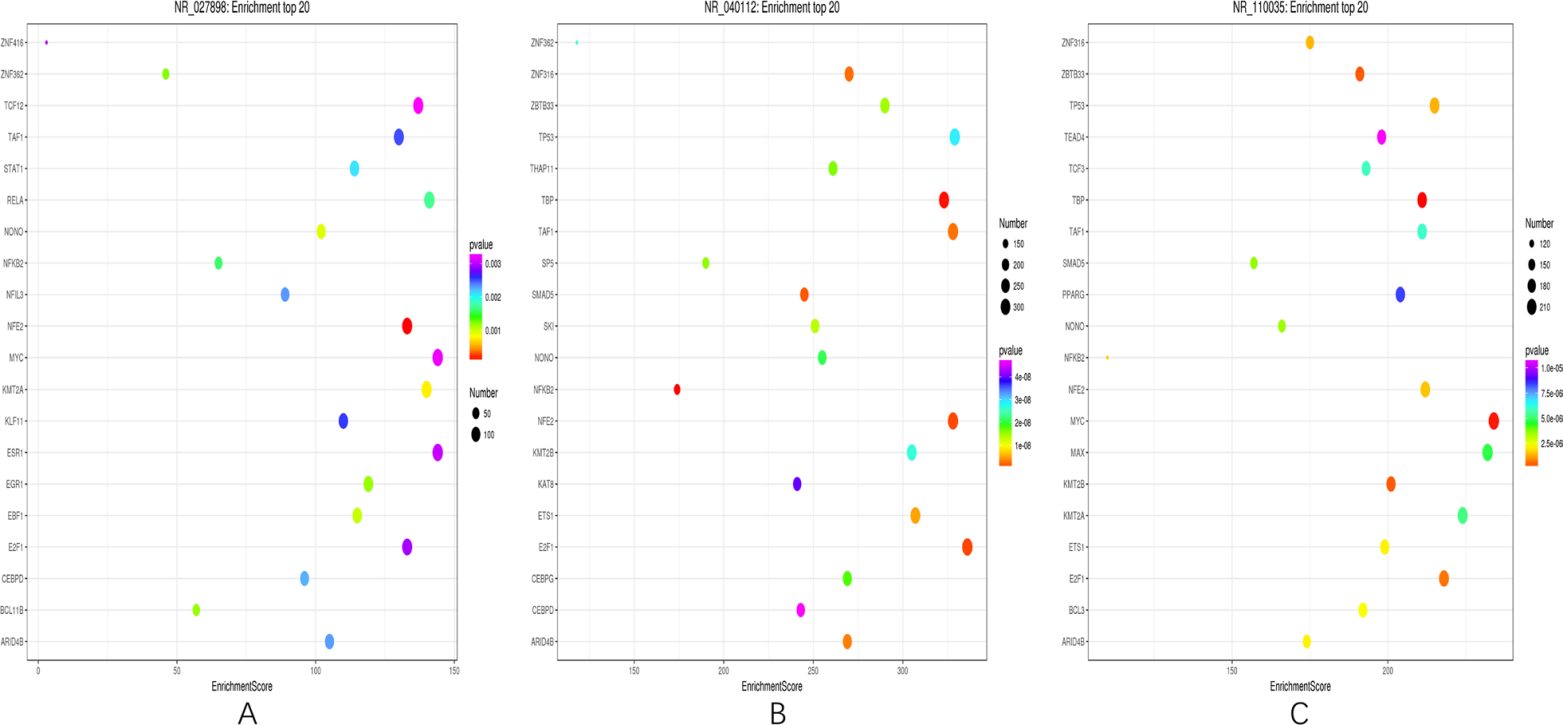
Predicted top 20 related transcription factors (TFs) of three differentially expressed lncRNAs including COL6A4P2 (**A**), A2MP1 (**B**), LOC100996671 (**C**). The x-axis represents the enrichment score, and the larger the bubble, the more differential coding genes it contains. The bubble color changes according to purple-blue-green-red. The smaller the enrichment *p* value, the greater the significance.

We predicted the potential TF targets of lncRNAs to dig the functions of Top 500 differentially expressed lncRNAs. After analysis, 122 differentially expressed lncRNAs corresponded to more than 80,000 TFs. For each lncRNA enriched TF, the first 20 items with the lowest P value were selected to draw a bubble chart, and the enriched results of 3 most differentially expressed lncRNAs (COL6A4P2, A2MP1, LOC100996671) were shown in Fig. 8A–C.

## Discussions

﻿The chronic symptomatic tendinopathy, such as LHBT tendinopathy, comprise a proportion of 30% to 50% during musculoskeletal and exercise-related problems [12, 26]. In addition, the function of LHBT with RCT and its role in anterior shoulder pain and disability have caused widespread controversy [27]. Currently, in most cases, anterior shoulder pain attributed to the biceps tendon does not seem to be caused by an inflammatory process. The histological findings of the extra-articular part and synovial sheath of LHBT are similar to the pathological findings of De Quervain’s tenosynovitis of the wrist, and may be due to similar chronic degenerative processes and other tendinopathy of the body [28]. Joseph et al. evaluated the intra-articular and extra-articular parts of the diseased LHBT and believed that the intra-articular part of LHBT showed many histological features of tendinopathy, while the structure of the extra-articular part was still similar to healthy tendons [29]. In addition, Zabrzyn ´ski et al. have conducted a series of studies on smoking and RCT and LHBT histopathology. They found that smoking was significantly associated with the occurrence of a large number of RCTs and the degree of pain by comparing smokers and non-smokers underwent shoulder arthroscopy due to complex LHBT pathology and RCTs. ﻿By the histopathologic evaluation of the harvested intra-articular portion of LHBT, they also presented an ambiguous role of the neovascularization in the biceps tendinopathy. The neovascularization process is crucial in biceps tendinopathy and was significantly reduced in patients with smoking history. Furthermore, the morphological alterations of rotator cuff tendons also correlated positively with the extent of biceps tendon degeneration [30, 31]. These studies indicated the influence of RCT on the structural and biochemical changes in LHBT. LncRNAs is a rising star in biology, and its regulatory function has been well confirmed in many diseases, and can be used to analyze the pathogenesis of this tendinopathy. RNA sequencing was used to detect the number of differentiation expressed mRNA and lncRNA in LHBT after RCT. The consistency of the four lncRNA expressions were confirmed by qRT-PCR. By further integrating our data, the potential regulatory mechanisms of these differentiation expressed lncRNAs and mRNAs were explored with bioinformatics methods. As far as we know, this study is the first study of lncRNA in LHB tendinitis after RCT.

Of the differentially expressed lncRNAs, 2 upregulated lncRNAs (LOC100996671, A2MP1) and 2 down-regulated ones (lnc-LRCH1-5, COL6A4P2) were verified and only A2MP1 had been reported in other disease, yet the other 3 lncRNAs are reported firstly. Among those differentiation expressed lncRNAs, several thoroughly studied molecules including A2MP1, C1QTNF1-AS1, CASC2, FTCDNL1, FTX, LOC339975 and TWIST1, which expressed in other diseases, were also significantly changed in LHBT. 50 SNPs were identified and evaluated for replication by Zeng et al. Through genome-wide association analysis, Rs16918212 located in A2MP1 was associated with cough in both the identification odds ratio and the meta-analyzed replication cohort [32]. Li et al. showed that C1QTNF1-AS1, who firstly down-regulated miR-221-3p and then up-regulated SOCS3, can inhibit the proliferation, migration and invasion of human liver cancer cells, and further accelerate apoptosis by acting on the JAK/STAT signaling pathway [33]. Zhang’s study concluded that CASC2 upregulation suppressed high glucose-induced proliferation, oxidative stress of human mesangial cells and extracellular matrix accumulation through miR-133b/FOXP1 regulatory pathway, suggesting that CASC2 was a novel biomarker for diabetic nephropathy treatment [34]. Lu’s findings proved that rs10203122 in FTCDNL1 have identified a link to a susceptibility to osteoporosis [35]. Modulating both microRNAs and gene expression, FTX may affect a lot in pathogenesis of rheumatoid arthritis, which as one of chronic inflammatory autoimmune disease [36]. LHB tendinitis is also a chronic inflammatory disease, so we deem that FTX may play an important role in LHBT pathogenesis. Adkins et al detected association between alcohol dependence and COL6A3, LOC339975, RYR3, and KLF12, and gene alteration in human nucleus accumbens could be influenced by the associated LOC339975 allele [37]. A vital function of TWIST1 in progenitors of human skeletal muscle was dug by whose critical role in maintenance of human putative skeletal muscle progenitor cells [38]. LHBT came from human putative skeletal muscle progenitor cells, so we hold that TWIST1 may affect a lot in the development of LHBT. Through GO analysis to annotate the biological processes of differentiation expressed lncRNA and mRNA, 10 most significant GO terms are linked to the innate immune response, neutrophil chemotaxis and the regulation of the cellular response to interleukin-1.

According to the results of GO analysis and KEGG pathway analysis related to immune diseases, the role of immune inflammatory response in LHB tendinitis and the role of lncRNAs in LHBT should be paid attention. Millar et al. found that tendon cells treated with IL-17A showed increased production of pro-inflammatory cytokines, altered matrix regulation, increased type III collagen, and increased expression of several apoptosis-related factors. They proposed that the IL-17 signaling pathway is an inflammatory mediator in the early process of tendinopathy, thus providing a new treatment method for the treatment of tendinopathy [39]. Here, our data shows that, at least in terms of the immune inflammatory response of LHBT, the expression and functional pattern of lncRNA may be similar to that of rheumatoid arthritis synovium, which may provide a new perspective for the diagnosis and treatment of LHB tendinitis.

﻿In this study, we found many dysregulated lncRNAs in LHBT after RCT, and we predicted their corresponding mRNAs through co‑expression network, cis-acting elements, trans-acting factors and TF enrichment. During trans-acting factors, we found that one lncRNA (PTPRG-AS1) can target many mRNAs, and some mRNAs (such as NQO1) seem to be regulated by varied lncRNAs, which shows the functional complexity of lncRNAs. In addition, NQO1 is one mediator of Nrf2/ARE signal pathway which taking part in regulation of inflammatory process. After Nrf2 gene is activated, Nrf2-ARE signal pathway is activated, which makes HO-1 gene, an important anti-inflammatory enzyme, be expressed, thus increasing the content of carbon monoxide and inhibiting the activity of macrophages, playing an anti-inflammatory role [40]. During TFs, lncRNA-COL6A4P2 targets STAT1, RELA, NFKB2, MYC. lncRNA-A2MP1 targets NFKB2, and lncRNA-LOC100996671 targets NFKB2, MYC. As is known to all, STAT1, the protein encoded by this gene is a member of the STAT protein family. Most cytokine receptors do not possess tyrosine kinase activity per se, can undergo dimerization upon binding to cytokines, and activate receptor associated Janus kinases (JAKs). Specific tyrosine residues on the receptor, when phosphorylated by JAKs, may provide binding sites for STAT in the cytosol. STAT after being phosphorylated by JAKs, can also form dimers, which translocate into the nucleus and activate related genes. MYC is a downstream mediator of the Jak / STAT signaling pathway, and it is an important regulatory mechanism that mediates various physiological and pathological responses. Inflammatory factors can activate JAK kinase and promote phosphorylation of stat, thereby causing inflammatory factor expression and cell damage, cell apoptosis or proliferation [41, 42]. De et al. revealed that up-expression of STAT1 leads to inflammation of STAT1-dependence, which described the underlying mechanism of inflammation of joint for myeloid-specific A20-deficient mice [43]. A subunit of the TF complex NF-κB was encoded by NFKB2. The expression of whom is exist in a variety of cell types and who as a central activator of genes, NF-κB complex regulates inflammation and immune function. NF-κB consists of NFKB1 or NFKB2 combined with REL, RELA or RELB. NFKB1 compounded with the gene product RELA is the most extensive form [44, 45]. For rheumatoid arthritis, Sabir's study used a weighted gene co-expression network to analyze the function of the NF-κB protein family and its regulators, and proved that these genes (such as NFKB2) may be involved in the inflammation and immune pathogenesis of rheumatoid arthritis with an important role [46]. Therefore, we screened these differentially expressed cis, trans, and TFs-acting lncRNAs, which act on genes related to LHB tendinitis. lncRNA-COL6A4P2, A2MP1 and LOC100996671 may affect the expression of STAT1, RELA, NFKB2 and MYC through the Jak/STAT axis and the NF-κB axis, thereby regulating the inflammatory response of LHBT. Nevertheless, basing on theoretical analysis, these hypothetical connections and interactions are feasible and require experiment validation. These bioinformatically predicted signal pathways perturbed by these lncRNAs would be validated using gene knockdown or siRNA technique in our following experiments.

In summary, we first constructed and analyzed the expression patterns of lncRNAs and mRNAs in LHB tendinitis after RCT. Bioinformatics analysis showed that differentiation expressed mRNAs and lncRNAs were mainly link to the regulation of immune inflammatory response. Some differentially expressed lncRNAs and their TF targets may provide new perspectives into the pathogenesis and may be promising approaches to analyze the gene pathomechanism of this inflammatory tendinopathy.

## Supplementary Information

**Additional file 1:** Real-time quantitative PCR primer sequences used in this study. PCR, polymerase chain reaction; bp, base pair.

## Data Availability

All sequencing data during this study had been deposited in NCBI SRA database. The project information will be accessible with the permanent link (http://www.ncbi.nlm.nih.gov/bioproject/780094) and the BioProject ID is PRJNA780094.

## Abbreviations

RCT: Rotator cuff tear
LHBT: Long head of biceps tendon
lncRNA: Long non-coding RNA
GO: Gene ontology
KEGG: Kyoto encyclopedia of genes and genomes
TF: Transcription factor
qRT-PCR: Real-time quantitative reverse transcription-polymerase chain reaction
GAPDH: Glyceraldehydes-3-phosphate dehydrogenase
BP: Biological process
CC: Cell component
MF: Molecular function
FDR: False discovery rate
JAKs: Janus kinases
