# Supplementary material for: mRNA and long non-coding RNA expression profiles of rotator cuff tear patients reveal inflammatory features in long head of biceps tendon
Source: BMC Med Genomics. 2022 Jun 20;15:140. doi: 10.1186/s12920-022-01292-y (PMC9210618; doi:10.1186/s12920-022-01292-y)
Supplement: Supplementary file 1 — Additional file 1: Real-time quantitative PCR primer sequences used in this study. PCR, polymerase chain reaction; bp, base pair. [file 12920_2022_1292_MOESM1_ESM.docx]

| **Additional file 1**. Real-time quantitative PCR primer sequences used in this study. | | | | |
| --- | --- | --- | --- | --- |
| ﻿Probe Name | ﻿Forward primers (5′-3′) | ﻿Reverse primers (5′-3′) | ﻿ | Amplicon size (bp) |
| A2MP1 | TGCCTGAGATATAACATCCTT | GTCCCCAGTATAACTAATGTTG |  | 141 |
| lnc-LRCH1-5 | TGGATAGGGCTCTGCATCTGTG | AATGACAATGCGTTTCACTCTCTT |  | 147 |
| LOC100996671 | CTTTTCTCGGGCCTTCTGAAC | GTAGAATATTATCCAGGGTCGCA |  | 112 |
| COL6A4P2 | ACCTAAGGGAGCAAGAGGATTTTC | CTCCAGATGACCCTGGGATTC |  | 107 |

PCR, polymerase chain reaction; bp, base pair.
